# A Molecularly Imprinted Polymer-Based Thermal Sensor for the Selective Detection of Melamine in Milk Samples

**DOI:** 10.3390/foods11182906

**Published:** 2022-09-19

**Authors:** Manlio Caldara, Joseph W. Lowdon, Jeroen Royakkers, Marloes Peeters, Thomas J. Cleij, Hanne Diliën, Kasper Eersels, Bart van Grinsven

**Affiliations:** 1 Sensor Engineering Department, Faculty of Science and Engineering, Maastricht University, 6200 MD Maastricht, The Netherlands; 2 School of Engineering, Newcastle University, Newcastle upon Tyne NE1 7RU, UK

**Keywords:** melamine, molecularly imprinted polymers (MIPs), milk, heat-transfer method (HTM), food adulteration testing, low-cost melamine detection

## Abstract

In recent years, melamine-sensing technologies have increasingly gained attention, mainly due to the misuse of the molecule as an adulterant in milk and other foods. Molecularly imprinted polymers (MIPs) are ideal candidates for the recognition of melamine in real-life samples. The prepared MIP particles were incorporated into a thermally conductive layer via micro-contact deposition and its response towards melamine was analyzed using the heat-transfer method (HTM). The sensor displayed an excellent selectivity when analyzing the thermal response to other chemicals commonly found in foods, and its applicability in food safety was demonstrated after evaluation in untreated milk samples, demonstrating a limit of detection of 6.02 μM. As the EU/US melamine legal limit in milk of 2.5 mg/kg falls within the linear range of the sensor, it can offer an innovative solution for routine screening of milk samples in order to detect adulteration with melamine. The results shown in this work thus demonstrate the great potential of a low-cost thermal platform for the detection of food adulteration in complex matrices.

## 1. Introduction

**Figure 1:**
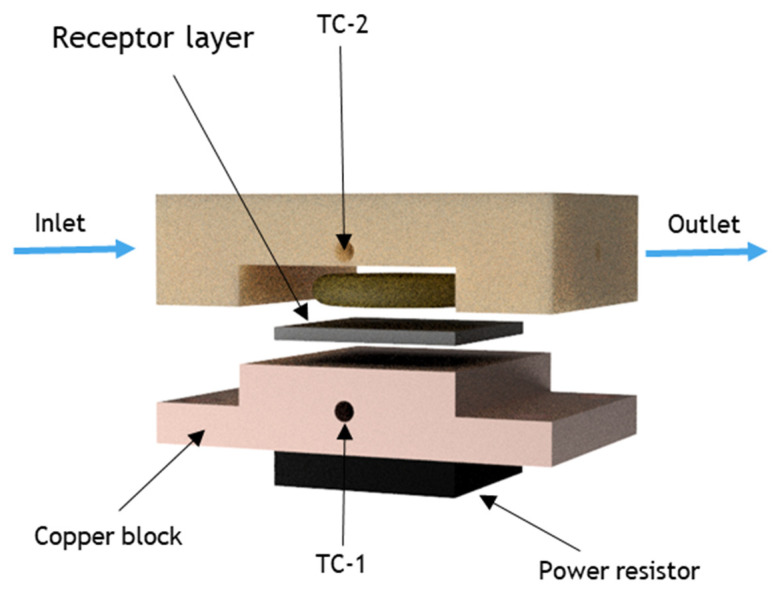
Graphical representation of the HTM analysis setup.

Melamine (C_3_H_6_N_6_) is a heterocyclic organic compound that is widely used in combination with formaldehyde and other agents to produce synthetic resins. Melamine resins are hard and highly durable materials, which find their application in the manufacture of many products, such as kitchenware and laminates [1]. Furthermore, because of its high nitrogen content (66% by mass), low price, and the fact that protein levels in milk are indirectly assessed via the total nitrogen content, it has been added intentionally into milk in order to mimic protein-rich milk products [2,3]. The oral ingestion of high concentrations of melamine can cause serious health issues, including bladder cancer [4], renal failure [5], or even fatality in humans and animals [6,7]. For this reason, the maximum residual levels allowed in milk and milk products are set at 2.5 mg/kg by The Codex Alimentarius Committee (CAC) [8]. Sensors that allow for routine screening of dairy products in a low-cost manner could enable end-users and industrial stakeholders at various points in the dairy production value chain so as to identify melamine adulteration rapidly and significantly decrease its adverse effects on the general healthcare system [9]. In the last few years, several well-established techniques have been developed for the quantification of melamine levels in milk products. However, most of these essays are based on expensive lab-based technologies such as high-performance liquid chromatography (HPLC) [10], liquid chromatography–tandem mass spectrometry (LC–MS/MS) [11,12], and surface-enhanced Raman scattering spectroscopy (SERS) [13,14]. Although current laboratory tests are very sensitive and can accurately determine the melamine levels in the samples, the analysis is typically slow and costly. Moreover, such complex matrices often require pre-treatment of the food sample to accurately perform these analyses [15,16]. As a result, standard practice is to only take samples at regulatory intervals and to screen them for melamine adulteration, leaving several cases unnoticed. Low-cost alternatives that allow for routine screening of products are thus highly desirable. Although low-cost melamine sensors are already present in the market, they usually do not offer quantifiable information in terms of the legal limit and they require reagents or labels. Moreover, these commercial sensors are based on enzymes, colloidal gold, or antibodies, with the main disadvantage for these being their limited stability, thus leading to relatively short shelf-life and the need for storing the sensor at determined temperatures and conditions. Biosensors could offer a low-cost alternative and in recent years, biosensors based on so-called molecularly imprinted polymers (MIPs) have specifically gained attention [17,18,19,20]. MIPs are synthetic receptors capable of selectively binding to a target through a “lock and key” mechanism and, as such, are considered synthetic substitutes of natural antibodies [21]. MIPs have been extensively studied in the last decades, and their high application potential in food safety and clinical diagnostics [22,23,24,25] is primarily due to the advantages over their natural counterparts in terms of chemical and environmental stability, straightforward preparation, and cost-effectiveness [26,27]. These features make MIPs ideal candidates for the recognition of different molecules and biomarkers in complex matrices [28,29,30]. To develop viable sensors, MIPs need to be combined with a readout technology that is capable of converting binding events at the receptor surface into a quantifiable signal, while operating in complex media. The heat-transfer method (HTM) [31] can detect temperature changes across a solid–liquid interface, allowing for various targets, ranging from small molecules to bacteria, and, more recently, viral antigens for COVID-19 diagnosis [32,33,34,35,36]. In short, when the target solution is introduced into the flow cell, MIP particles bind to the analyte, resulting in a thermal change at the interface. This change is then detected as a change in temperature in the medium above a sensor chip, registered by a thermocouple placed inside the flow cell (TC-2), while another thermocouple (TC-1), coupled to a PID-based temperature control system, stringently steers the input temperature underneath the sample (Figure 1). By actively steering the temperature input and monitoring the temperature output, the sensor is able to monitor the difference in thermal resistance across the solid–liquid interface.

This study aims to prove that an HTM-coupled, MIP-based melamine receptor can form the basis for routine analysis and a low-cost adulteration sensor in untreated food samples. Therefore, in this work, MIPs were synthesized and optimized for the detection of melamine by varying the recipe composition. The rebinding performance of each of the resulting MIPs was analyzed using UV–VIS spectroscopy. The analysis of the rebinding capabilities and the imprinting efficiency of the different recipe compositions permitted the selection of the best MIP ratio, which was then used throughout the study for all of the HTM measurements. The optimized MIP particles were then integrated into a sensing chip via micro-contact deposition on a polyvinylchloride-covered aluminum HTM substrate. The resulting sensor illustrated the specificity and reproducibility of the MIP-platform for the detection of melamine in CaCl_2_ when compared with the corresponding NIP. The selectivity of the layer was established by comparing the thermal response of the receptor layer to the target and molecules that are commonly found in milk samples (cyanuric acid and lactose) or other contaminants found in different foods and beverages (bisphenol A). To demonstrate the application of the sensor in food safety analysis, it was exposed to known concentrations of melamine in untreated milk samples, and the thermal response was recorded and evaluated. The resulting linear range and LoD were compared with the legal limit in EU and USA, proving the applicability of the MIP-based platform for the monitoring and detection of melamine levels in food samples.

## 2. Materials and Methods

### 2.1. Chemicals and Reagents

Melamine (99%), D-lactose monohydrate (≥99.5%), bisphenol A (≥99%), polyvinyl chloride (MQ200), acetic acid (99.8%), calcium chloride (≥97%), methacrylic acid (99.5%), tetrahydrofuran (99.5%), dimethyl sulfoxide (99.7%), and 2,2′-azobis(2-methylpropionitrile) (98%) were purchased from Sigma-Aldrich Chemie B.V. (Zwijndrecht, the Netherlands). Ethylene glycol dimethacrylate (98%) and methanol (≥99.9%) were obtained from TCI Chemicals. Cyanuric acid (99%) and ethanol (96%) were purchased from Fisher Scientific (Landsmeer, the Netherlands). Prior to polymerization, functional and cross-linking monomers were passed over an aluminum-oxide-packed column to remove stabilizers. All stock solutions were prepared with Milli-Q water of 18.2 MΩ cm^−1^. Polydimethylsiloxane (PDMS) stamps were made using the Sylgard 184 elastomer kit supplied by Mavom N.V. (Schelle, Belgium). Aluminum chips were obtained from Brico N.V. (Korbeek-Lo, Belgium) and cut to 1 cm^2^ surfaces. The melamine rapid test kit was purchased from Ballya Bio-Med Co., Ltd. (Guangzhou, China).

### 2.2. Synthesis of Molecularly Imprinted Polymers

The ratios and procedures employed for the synthesized MIPs were based on previous literature regarding the monolithic polymerization of molecularly imprinted polymers for other targets [37,38]. The best rebinding efficiency in this study was obtained with a 1:14:28 ratio of template/monomer/cross-linker. In short, MEL template (0.25 mmol, 31.5 mg), MAA functional monomer (3.5 mmol, 297 μL), EGDMA cross-linker (7 mmol, 1.32 mL), and AIBN initiator (0.24 mmol, 40 mg) were dissolved in dimethyl sulfoxide (DMSO; 5 mL). The reaction mixture was bubbled with N_2_ for 20 min to remove the oxygen before polymerization. Next, the mixture was heated to 65 °C for 8 h. The resulting bulk MIP were then ground into finer particles and washed with methanol to remove unreacted reagents. The MIP solids were then collected by filtration and dried in an oven for 12 h at 65 °C. The dried particles were milled three times using a Fritsch Planetary Micro Mill Pulverisette7 premium line (300 rpm, 3 min, and 10 mm balls), after which the powder was collected and stored. Finally, melamine was extracted from the MIP via Soxhlet extraction (1:10 mixture of DI water to ethanol) for 16 h, and subsequently methanol for another 16 h. The extraction cycles were repeated until no template molecule was detected via UV–VIS spectroscopy. The extracted particles were then dried at 65 °C for 12 h. A reference NIP was prepared in parallel following the same procedure, but in the absence of the analyte.

### 2.3. Fourier Transform Infrared Spectroscopy

Complete template extraction was confirmed through comparative IR spectroscopic analysis of the extracted MIP and non-extracted MIP and NIP samples, using an IR Spirit Fourier transform infrared (FT-IR) spectrometer (Shimadzu Corp., Kyoto, Japan) set at 64 scans and 8 cm^−1^ spectral resolution per measurement, with a spectral range of 4000 to 400 cm^−1^.

### 2.4. Batch Rebinding Experiments

Optical batch rebinding experiments were recorded with a Shimadzu UV-1900i spectrophotometer. To establish the binding affinity of the MIP/NIP, 20 mg of MIP/NIP particles were incubated with 5 mL solutions of melamine in Milli-Q water, with a concentration range from 0.02 to 0.5 mM. The samples were then shaken at 130 rpm for 90 min. The suspensions were then left to settle, after which the filtrate was collected and analyzed using a UV–VIS spectrophotometer. The remaining unbound concentration of melamine (C_f_) in solution was then determined by UV–VIS spectroscopy, using a calibration curve for melamine that was generated prior at λ max (235 nm) (Figure S1).

### 2.5. Preparation and Characterization of MIP-Based Receptor Layer

The MIP-based receptor layer was prepared according to previous literature, with slight modifications [39]. First, a polyvinyl chloride (PVC) adhesive layer was deposited on the aluminum chip by spin coating (1000 rpm for 60 s with an acceleration of 1000 rpm s^−1^) a 2.0 wt% PVC solution in tetrahydrofuran. Next, a PDMS substrate (1 cm^2^), covered with a monolayer of MIP particles, was used to stamp the particles into the PVC layer. The PVC layer was heated to above the glass transition temperature (100 °C), allowing the particles to settle into the polymer layer. After cooling, the unbound particles were washed away with DI water before thermal measurements.

The developed platform was characterized by analyzing the sensor’s surface prior to and after deposition of the MIP particles onto the Al-PVC substrate. To this end, the samples were punched into 12 mm disks and after gold coating, the samples were imaged using a Jeol JSM-IT200 InTouchScope scanning electron microscope (JEOL, Peabody, MA, USA) at V = 10 keV. For analysis of the chemical composition of the Al-PVC layer and of the deposited MIP particles, the samples were then studied under an energy dispersive X-ray analyzer (EDX) coupled to the Jeol JSM-IT200 microscope (see Figure S6).

### 2.6. Heat-Transfer Sensing Setup

The thermal detection platform has been described in detail in previous work [40]. In short, the MIP-coated aluminum substrates were attached to a copper heat sink. The temperature of the heat sink, T1, was stringently controlled using a K-type thermocouple (TC Direct, Nederweert, the Netherlands), a power resistor (Farnell, Utrecht, the Netherlands), and a software-based proportional-integral-derivative (PID) controller (P = 10, I = 8, D = 0) in Labview (National Instruments, Austin, TX, USA). The MIP layer was exposed to the samples under analysis by means of an injection molded polycarbonate (PC) flow cell (A = 28 mm^2^, V = 110 μL). Samples were injected into the flow cell by means of a syringe pump at a flowrate of 0.250 mL/min for 5 min. To monitor the thermal resistance at the solid–liquid interface (the MIP-functionalized surface), a second thermocouple monitored the temperature inside the flow cell, T2, at a constant input temperature of 37.00 °C. To mimic milk, CaCl_2_ solutions (1.6 mM) were used as buffer solutions to stabilize the signal. Gradually, the melamine or analogue concentration in buffer (or milk for the real sample analysis) was increased within the regulatory relevant concentration regime (15–90 μM). The signal was allowed to stabilize for 20 min between each addition. The specificity was assessed by comparing the MIP response to that of the NIP reference, while the selectivity was examined using cyanuric acid, bisphenol A, and lactose as the analogue molecules.

### 2.7. Thermal Detection of Melamine in Milk Samples

The absence of melamine in the analyzed milk samples was confirmed using a commercial melamine rapid test kit. Afterwards, the untreated milk samples were spiked with increasing melamine concentrations, and the obtained solutions were employed for thermal analysis using the HTM setup.

## 3. Results

### 3.1. Batch Rebinding via UV–VIS

Four MIP compositions were synthesized and their capacity to specifically bind melamine in aqueous samples was assessed (Table 1). All of the compositions were prepared with methacrylic acid (MAA) as a functional monomer in order to create ionic and hydrogen bonds with the amine functionalities present in the melamine molecule (Figure S2). Ethylene glycol dimethacrylate (EGDMA) and 2,2′-azobis(2-methylpropionitrile) (AIBN) were used as the cross-linker molecule and thermal initiator, respectively. After synthesis, the MIPs were washed and the template was extracted by means of Soxhlet extraction, with template removal being confirmed by FT-IR analysis (Figure S3).

**Figure 2:**
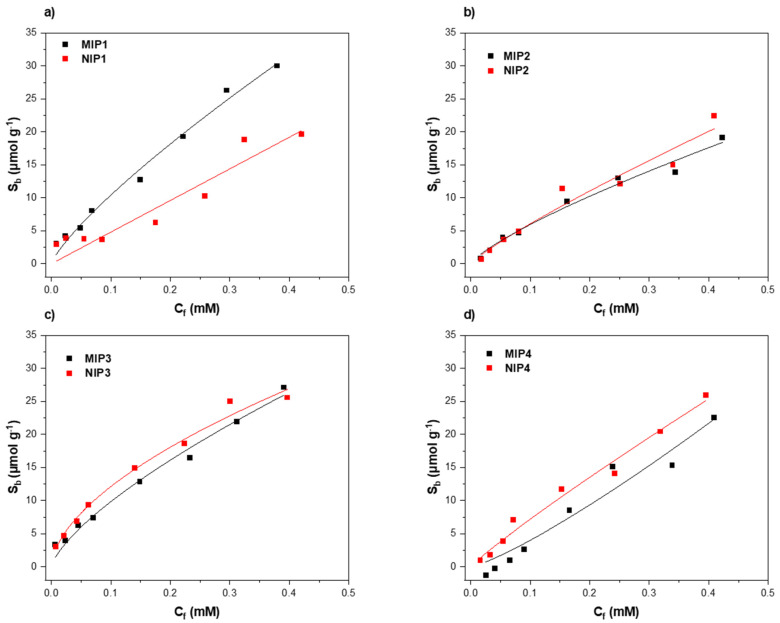
Rebinding analysis with UV–VIS spectroscopy of (**a**) MIP/NIP1, (**b**) MIP/NIP2, (**c**) MIP/NIP3, and (**d**) MIP/NIP4.

For each composition, a dose–response curve was constructed by calculating the free concentration of melamine found in the solution (C_f_) (mM) after the rebinding experiment, and to obtain the corresponding substrate bound (S_b_) (μmol g^−1^) values, which indicate the amount of target bound (in micromoles) per grams of MIP at each data point. Finally, the obtained C_f_ and S_b_ values were plotted and dose–response curves for all of the synthesized MIP/NIP compositions were obtained. The data were fitted (allometrically; y = ax^b^) with Origin 2021b (OriginLabs Corporation, Northampton, MA, USA). In Figure 2, each of the synthesized MIP compositions (black squares) are plotted together with the corresponding NIP (red squares).

Of the examined formulations, MIP-2 displayed the lowest overall maximum binding capacity with a maximum S_b_ of 19.12 μmol g^−1^, and in contrast, MIP-1 had the highest maximum binding capacity (max S_b_ = 30.00 μmol g^−1^), thus demonstrating that higher concentrations of functional monomer (MAA) and cross-linker (EGDMA) are needed to generate a higher number of nano-cavities in the polymer. When comparing MIP-2 and MIP-3, it should be noted that with a higher concentration of monomer (MIP-3), a higher binding capacity was obtained. However, it was surprising that MIP-4 exhibited a slightly lower binding capacity than MIP-3 (22.53 and 27.11 μmol g^−1^). MIP-4 was prepared with the same monomer:cross-linker ratio as MIP-3, but with doubled equivalents with respect to the template, thus showing that doubling the concentration of monomer and cross-linker but maintaining a constant monomer:crosslinker ratio led to a similar maximum binding capacity. In order to comprehensively scrutinize the specific binding per MIP/NIP and to compare the performance of MIPs made with different recipes, the imprinting factor (IF) was calculated for each composition. The IF value is defined as the S_b_ value at a defined C_f_ for MIP divided by the S_b_ value of the corresponding NIP at the same C_f_, and is a measure of the specificity of the developed MIP system. To obtain a more accurate comparison between the different formulations, C_f_ values at 0.1 mM were selected to calculate the IF values for each MIP/NIP (Table 1). When analyzing the IF of the four compositions, it was clear that MIP-1 demonstrated the best specificity with an IF of 2.22. When analyzing the other MIP compositions (MIP-2, MIP-3, and MIP-4), these showed a slightly lower maximum binding capacity, as per the one calculated for MIP-1, which could mainly be attributed to a higher degree of non-specific binding, demonstrated by the high S_b_ values of the corresponding NIPs. This resulted in IF values ranging from 0.56–0.96, thus showing that most of the improvement in the binding capacity could be attributed to an increase in non-specific binding sites, which was also confirmed by the fact that the corresponding NIPs had an even higher S_b_ value than the corresponding MIPs. Therefore, when considering both the maximum binding capacity and the IF values for each of the formulations, it was clear that the MIP that should be used for the remainder of the experiments was MIP-1.

### 3.2. Rebinding Analysis Using HTM

**Figure 3:**
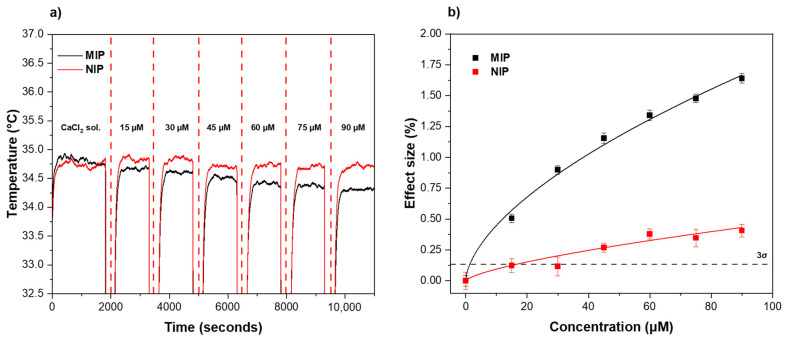
(**a**) Temperature profiles and (**b**) dose–response curves (from HTM analysis) of the MIP/NIP-based receptor layer after injections with increasing concentrations of melamine (0–90 μM) in CaCl_2_. The LoD (3σ method) of ±1.45 μM is indicated by the black dashed line. Triplicate measurements were used to calculate the error bars and mean values.

After preparation of the receptor layer via micro-contact deposition, the thermal response of the functionalized aluminum surfaces to melamine was examined using the HTM method. The receptor layer was therefore exposed to different concentrations of melamine (0–90 μM) in 1.6 mM CaCl_2_ solutions over a defined time period (Figure 3). To enable direct comparison between MIPs and NIPs, the analysis was run in parallel. A distinct decrease in temperature within the flow cell was observed when a higher concentration of melamine was introduced across the MIP-based receptor layer (black line). This trend was traced back to the interactions between the MIP particles and the analyte. More specifically, MIP contained recognition sites that were complementary to the melamine molecule in the shape, size, and distribution of the functional groups, thus allowing the analyte to perfectly fit in the nano-cavities present in the polymeric network. When the binding event occurred, the MIP cavities at the surface of the receptor layer were gradually filled, changing the thermodynamic properties of the interfacial layer, which translated into an increase in the interfacial thermal resistance, registered as a diminished heat flow through the sample into the solution inside the flow cell (Figure 3a, black line). In comparison, the same effect could be observed when analyzing the thermal response of the NIP (Figure 3a, red line), but the decrease in temperature in the flow cell in response to higher concentrations of melamine was much lower. This trend was mainly attributed to non-specific interactions between melamine and functional groups on the NIP surface. The temperature profiles over time were used to construct dose–response curves for both MIP and NIP by plotting the different concentrations of melamine injected against the effect size as a function of the temperature change. The effect size was calculated using the same equation reported in previous work [39] (Equation (1)).

(1) $$\mathrm{Effect}\;\mathrm{size}\;\left(\%\right)=\frac{\Delta T}{T_{{\mathrm{CaCl}}_{2}}}\times 100$$

In short, the value was obtained by dividing the temperature decrease after each injection by the average baseline temperature obtained after stabilization in CaCl_2_. An allometric (y = ax^b^) fit was used for both the MIP (black curve, R^2^ = 0.9891) and NIP (red curve, R^2^ = 0.8638) using Origin, version 2021b (OriginLab Corporation, Northampton, MA, USA). From the dose–response graph, the LoD of the MIP (black dashed line) was calculated using the 3σ method, corresponding to the concentration at which the effect size became greater than three times the maximum noise on the temperature signal (y-axis) of the measurement. The LoD was obtained by calculating the 3σ y-value and drawing a horizontal line on the curve at this y-value. The intercept of the 3σ line with the linear part of the MIP curve showed a LoD for the sensor equal to 1.45 μM. The capability of the sensor to bind melamine in a specific manner was demonstrated by analyzing the intercept for the LoD for the MIP with the corresponding intercept for the NIP curve, the latter resulting in a significantly higher LoD value. Moreover, the sensor demonstrated a strong linear trend with little saturation effect when exposed to concentrations ranging from 0 to 90 μM. The reference NIP, instead, showed saturation at low concentrations and had a clearly lower effect size when compared with the MIP.

### 3.3. Selectivity Analysis of the Receptor Layer

**Figure 4:**
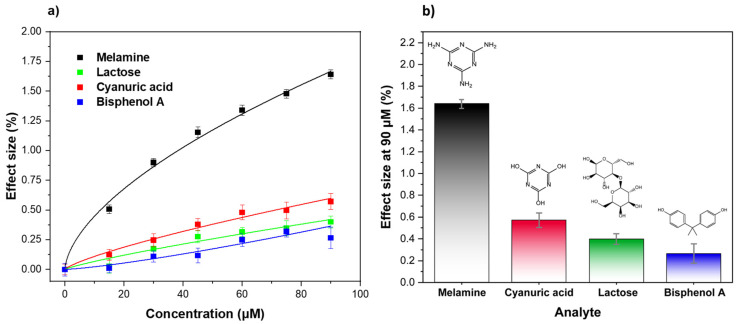
(**a**) Dose–response curves obtained via HTM analysis of different molecules (melamine, cyanuric acid, lactose, and bisphenol A) and (**b**) comparison of the calculated effect sizes at 90 μM. Triplicate measurements were used to calculate the error bars and mean values.

To determine the selectivity, the functionalized receptor layer was exposed to different analogue molecules, commonly found in contaminated milk and other foodstuffs (e.g., cyanuric acid, bisphenol A, and lactose) and the sensor’s response was recorded via HTM thermal analysis (Figure 4). The same concentration ranges (0–90 μM) were employed and the temperature profiles were converted into an effect size (%) using Equation (1). As observed, the affinity of MIP towards melamine was much greater than for the structural analogues (Figure 4a), already demonstrating a clear difference from the first analyte injection (15 μM). The effect size increased with increasing the analyte concentrations, demonstrating a much higher specificity of the sensor toward melamine at higher concentrations. Figure 4b shows the difference in effect size between melamine and the other tested compounds at 90 μM, demonstrating that the thermal readout was also able to selectively recognize melamine over structural analogues at higher concentrations.

In order to provide a more comprehensive investigation of the selectivity of the sensor platform, the selectivity factor was calculated (Table 2) for all of the analogues under study. In short, the values were calculated by dividing the effect size values obtained after exposure of the sensor to the target molecule, using the values recorded after infusion with the same concentrations of the tested analogues. The selectivity factor was calculated at a concentration of 2.5 mg/kg. This concentration represents the legal limit of melamine in milk samples, and thus provides a key indication of the selectivity of the sensor in a real-word sample analysis. It can be seen that MIP revealed an effect size 3.62 times greater than the one recorded for its most similar structural analogue—cyanuric acid. The selectivity factor is even bigger for the other analogue molecules, which demonstrates that the structural difference between the analogue and target plays a key role during the recognition/binding event and that the presence of these molecules in food samples (e.g., milk) would not affect the reliability of the HTM-based sensor.

### 3.4. Real-Life Sample Analysis: Detection of Melamine in Milk Samples

**Figure 5:**
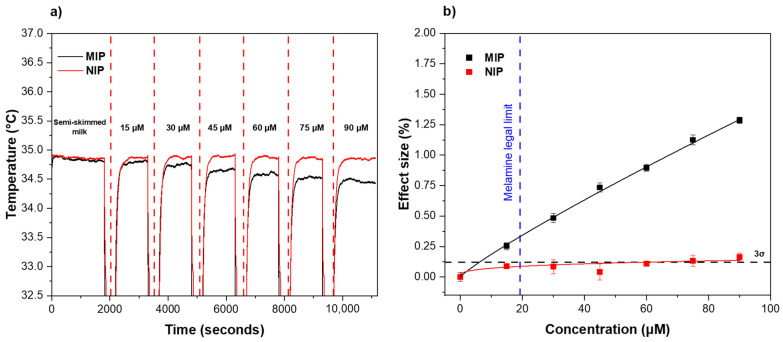
(**a**) Temperature profile and (**b**) dose–response curve obtained by an HTM analysis of the MIP/NIP-based receptor layer after infusions of different concentrations of melamine in spiked semi-skimmed milk samples. The black dashed line shows the LoD (3σ method) at ±6.02 μM. The blue dashed line indicates the legal limit of melamine in milk set by EU/US regulators. Triplicate measurements were used to calculate the error bars and mean values.

To establish the applicability of the sensor as a tool for identifying illegal melamine food adulteration, the thermal variations of the receptor layer were recorded and analyzed while being exposed to increasing concentrations of melamine in untreated, semi-skimmed milk samples (Figure 5a,b). The same experimental setup and parameters as per the rebinding analysis in CaCl_2_ were used for the investigation of melamine levels in milk. The absence of melamine in the non-spiked milk samples was confirmed with a commercially available colloidal gold immunochromatography assay for rapid melamine detection (Figure S5). To accurately compare the thermal measurement recorded in CaCl_2_ solution with the one obtained in milk samples, the milk samples were spiked to reach the same melamine concentration range (15–90 μM) as for the CaCl_2_ solution. Both the temperature profiles and the extrapolated dose–response curves for MIP and NIP showed a similar trend to the one obtained in the buffer solutions (Figure 3 and Figure 5). The LoD was 6.02 μM. The data obtained in milk samples display a comparable trend with the experiments performed in CaCl_2_ both in terms of rebinding efficiency and sensitivity, demonstrating that the sensor platform retained a high specificity in more complex real-life samples, such as untreated semi-skimmed milk. When comparing the LoDs in the different matrices, it was unsurprising that the sensor was less sensitive when employed directly in milk samples as a result of the complexity of the matrix employed. As can be seen in Table 3, our sensor demonstrated a higher LoD when compared with other potentially interesting new sensor platforms reported in the literature in recent years. However, the LoD in milk samples was still three times lower than the established legal limit of melamine in milk (blue dashed line) and therefore demonstrated the sensor’s potential as a novel low-cost tool to routinely screen milk samples without any pretreatment needed.

## 4. Conclusions

This study demonstrates the preparation of molecularly imprinted polymers for the detection of melamine in both artificial and real-life milk samples using thermal analysis. Out of all formulations, MIP-1 evidently performed the best in terms of both specificity and rebinding capacity (IF = 2.22 and Sb = 30 μmol g^−1^). Using the HTM method, the sensitivity of the MIP-based sensing platform in CaCl_2_ solutions was evaluated, showing a linear range of 1.45–90 μM and a LoD of 1.45 μM in the thermal data. Furthermore, the sensor proved to be highly selective towards melamine in comparison with structural analogues, milk components, and other contaminants found in beverages, demonstrating the efficiency of this platform in analyzing untreated, real-life samples. In fact, the obtained results in milk showed a linear range of 6.02–90 μM and a LoD of 6.02 μM, demonstrating the potential of the sensor in food analysis. The combination of an easily scalable production process with a cost-effective readout technology make these results very appealing for commercial applications, and will stimulate further development towards its integration into handheld devices.

## Figures and Tables

**Table 1:** Synthesized MIP/NIP compositions.

MIP/NIP	Melamine (mg)	MAA (eq.)	EGDMA (eq.)	AIBN (mg)	DMSO (mL)	R^2^	Max S_b_ (μmol g^−1^)	IF (at C_f_ = 0.1 mM)
**MIP1**	31.5	14	28	40	5	0.9882	30.00	2.22
**NIP1**	-	14	28	40	5	0.8977	19.66	
**MIP2**	31.5	3	20	40	5	0.9805	19.12	0.96
**NIP2**	-	3	20	40	5	0.9529	22.44	
**MIP3**	31.5	6	20	40	5	0.9848	27.11	0.82
**NIP3**	-	6	20	40	5	0.9870	25.55	
**MIP4**	31.5	12	40	40	5	0.9460	22.53	0.56
**NIP4**	-	12	40	40	5	0.9829	25.94	

**Table 2:** Selectivity factors of the developed thermal platform.

Substance	Selectivity Factor
**Cyanuric acid**	3.62
**Bisphenol A**	12.14
**Lactose**	5.76

**Table 3:** Comparison of recently developed sensors for melamine detection in food samples.

Readout Technology	Limit of Detection	Real Sample Analysis	Sample Pretreatment	Reference
**Differential pulse voltammetry (DPV)**	8.21 × 10^−^^12^ M	Liquid milk	Pretreatment needed	[41]
**Colorimetric assay (UV–VIS)**	0.099 μM	Raw milk	Pretreatment needed	[42]
**Surface-enhanced Raman spectroscopy (SERS)**	0.012 mmol L^−1^	Whole milk	Pretreatment needed	[43]
**Quartz crystal microbalance (QCM)**	2.3 ng mL^−1^	Liquid milk	Pretreatment needed	[44]
**Surface-enhanced Raman spectroscopy (SERS)**	0.1 ppm	Milk powder	Pretreatment needed	[14]
**Liquid Chromatography–Tandem Mass Spectrometry** **(LC–MS/MS)**	0.02–0.05 mg/kg	Egg powder, soy protein	Pretreatment needed	[11]
**Heat-Transfer Method (HTM)**	6.02 μM	Whole milk	No pretreatment needed	This work

## Data Availability

Data is contained within the article and Supplementary Materials.

## Supplementary Materials

The following supporting information can be downloaded at: https://www.mdpi.com/article/10.3390/foods11182906/s1, Figure S1: Calibration curve of melamine at 235 nm obtained via UV–VIS analysis; Figure S2: Chemical structures of template (melamine), functional monomer (methacrylic acid), and cross-linker (ethylene glycol dimethacrylate) employed to obtain the MIP particles used throughout the study; Figure S3: FT-IR analysis of the NIP, extracted MIP, and non-extracted MIP; Figure S4: Chemical structures of the tested competitive analytes; Figure S5: Confirmation that melamine is absent or present in trace amounts using a commercially available colloidal gold immunochromatography assay; Figure S6: SEM images of Al-PVC layer and MIP-based receptor layer. EDX analysis of Al-PVC layer and deposited MIP particle. References [45,46] are cited in supplementary materials.
