# Supplementary material for: A Molecularly Imprinted Polymer-Based Thermal Sensor for the Selective Detection of Melamine in Milk Samples
Source: Foods. 2022 Sep 19;11(18):2906. doi: 10.3390/foods11182906 (PMC9498381; doi:10.3390/foods11182906)
Supplement: Supplementary file 1 [file foods-11-02906-s001.zip › foods-1863778-supplementary.pdf]

A Molecularly Imprinted Polymer-Based Thermal Sensor for the Selective Detection of Melamine in Milk Samples

Manlio Caldara [1]*, Joseph W. Lowdon [1], Jeroen Royakkers [1], Marloes Peeters [2], Thomas J. Cleij [1], Hanne Diliën [1], Kasper Eersels [1] and Bart van Grinsven [1]

[1] Sensor Engineering Department, Faculty of Science and Engineering, Maastricht University, 6200 MD Maastricht, The Netherlands
[2] School of Engineering, Newcastle University, Newcastle upon Tyne NE1 7RU, UK

* Correspondence: m.caldara@maastrichtuniversity.nl

Supplementary figures:

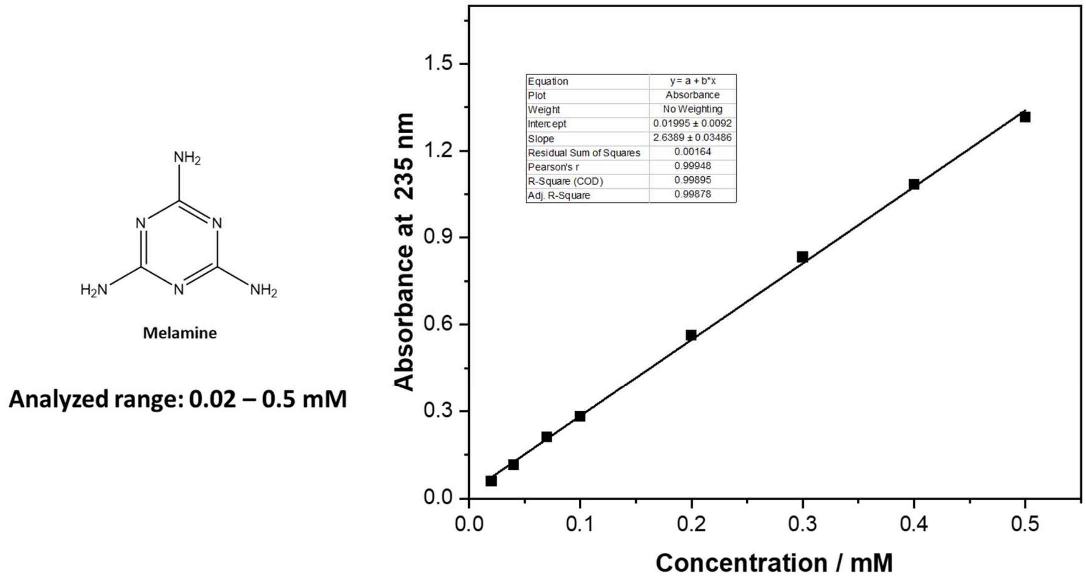

Figure S1. Calibration curve of melamine at 235 nm obtained via UV–VIS analysis.

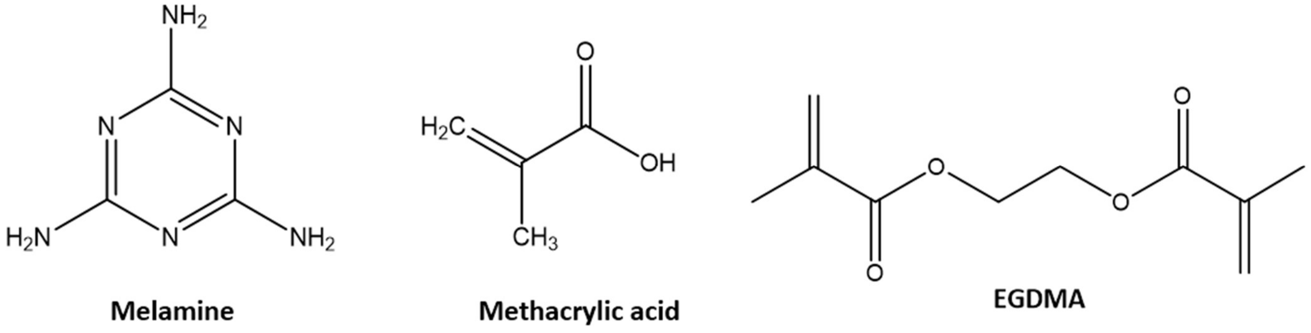

Figure S2. Chemical structures of the template (melamine), functional monomer (methacrylic acid), and cross-linker (ethylene glycol dimethacrylate) employed to obtain the MIP particles used throughout the study.

Analysis of Template extraction

The confirmation of the removal of the template from an MIP is crucial in order to obtain the best rebinding efficiency [1]. If the template molecule is not completely extracted from the polymeric network, less cavities are available for the rebinding, resulting in an inevitable reduced efficiency. To ensure the complete extraction of melamine from the imprinted polymer, FT-IR analysis was performed. As melamine has a low solubility in different solvents, several extractions with a Soxhlet apparatus were performed and a UV–VIS spectrum of the obtained filtrate was recorded after each cycle until complete extraction. To prove the successful removal of melamine from the polymeric network and thus the formation of nano-cavities in the extracted MIP, FT-IR analysis of non-extracted MIP, non-attracted MIP, and NIP were performed (Figure S3). In the FT-IR spectrum of the non-extracted MIP (red line), two distinctive peaks at 3440 and 3400 cm^{-1} and at 1015 and 1027 cm^{-1} can be observed; however, instead, they were absent in the NIP and extracted MIP spectra (blue and green lines). The two peaks are considered characteristic peaks of melamine and are assigned to the -NH2 stretch (typical of the melamine) and to the C-N stretching typical of primary amines, tertiary C [2]. Considering the FT-IR results, it can be stated with confidence that no significant presence of melamine was found in the imprinted polymer after Soxhlet extraction.

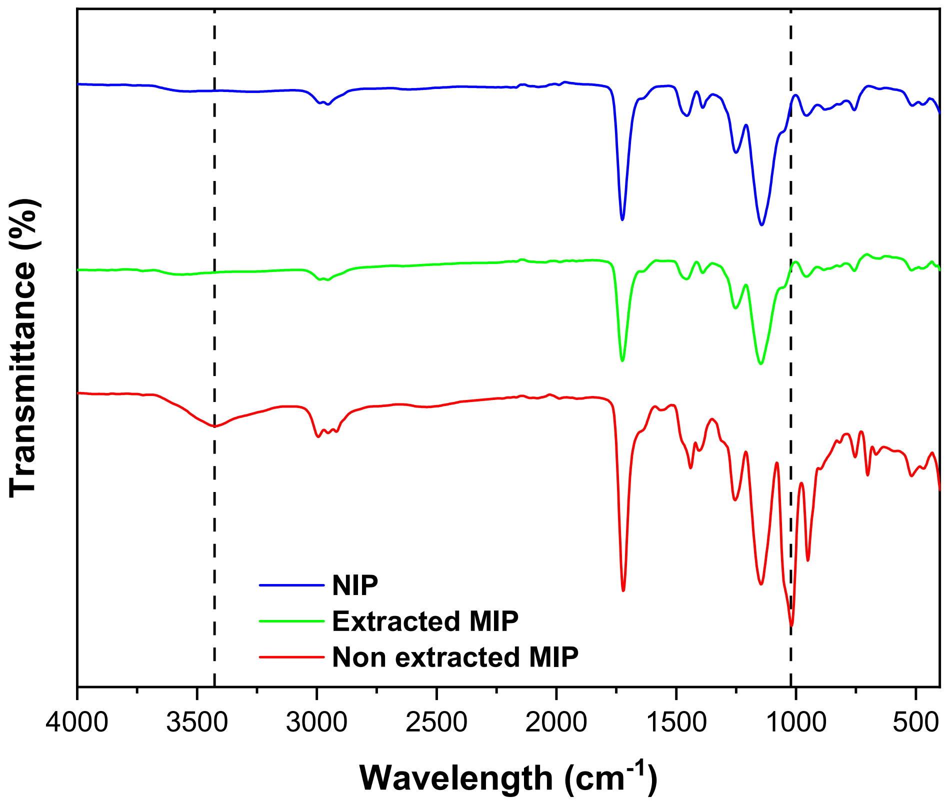

Figure S3. FT-IR analysis of the NIP, extracted MIP, and non-extracted MIP.

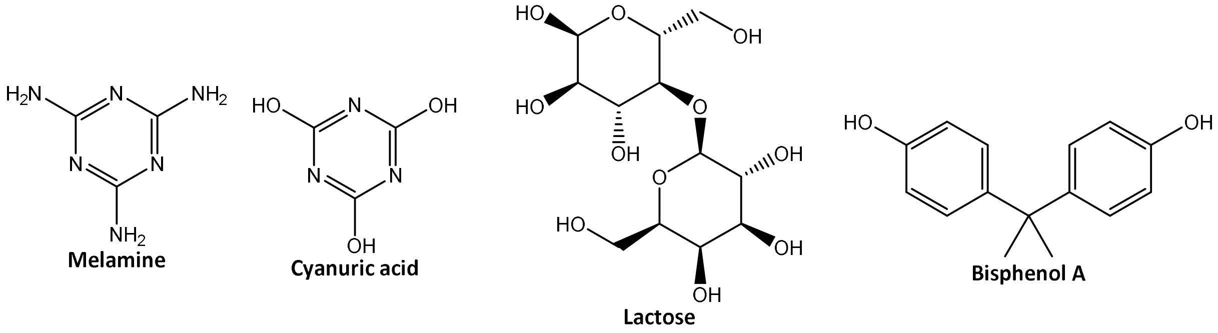

Figure S4. Chemical structures of the tested competitive analytes.

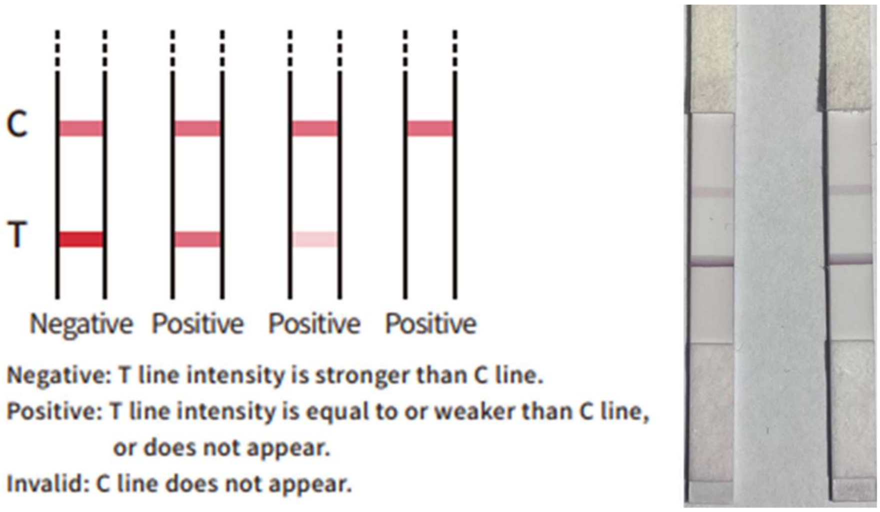

Figure S5. Confirmation that melamine is absent or present in trace amounts using a commercially available colloidal gold immunochromatography assay.

Characterization of MIP-based receptor layer

In order to characterize and demonstrate the successful deposition of MIP particles onto the Al-PVC substrate, SEM and EDX analysis of the receptor layer prior to and after deposition were performed (Figure S6). When comparing the SEM images of the Al-PVC layer before and after deposition, it is evident that a large amount of imprinted microparticles were deposited onto the substrate (Figure S6C), while in Figure S6A, a smooth layer onto the aluminum substrate can be observed. An examination of the chemical properties of the Al-PVC layer via EDX analysis (Figure S6B) shows the presence of Al, C, O, and Cl, confirming the formation of the PVC layer onto the Al substrate. Instead, when analyzing the deposited MIP particle (Figure 6D), the prevalence of the elements C and O can be clearly noticed. This result can be expected considering the chemical structures of monomer and cross-linker used to produce the imprinted polymer.

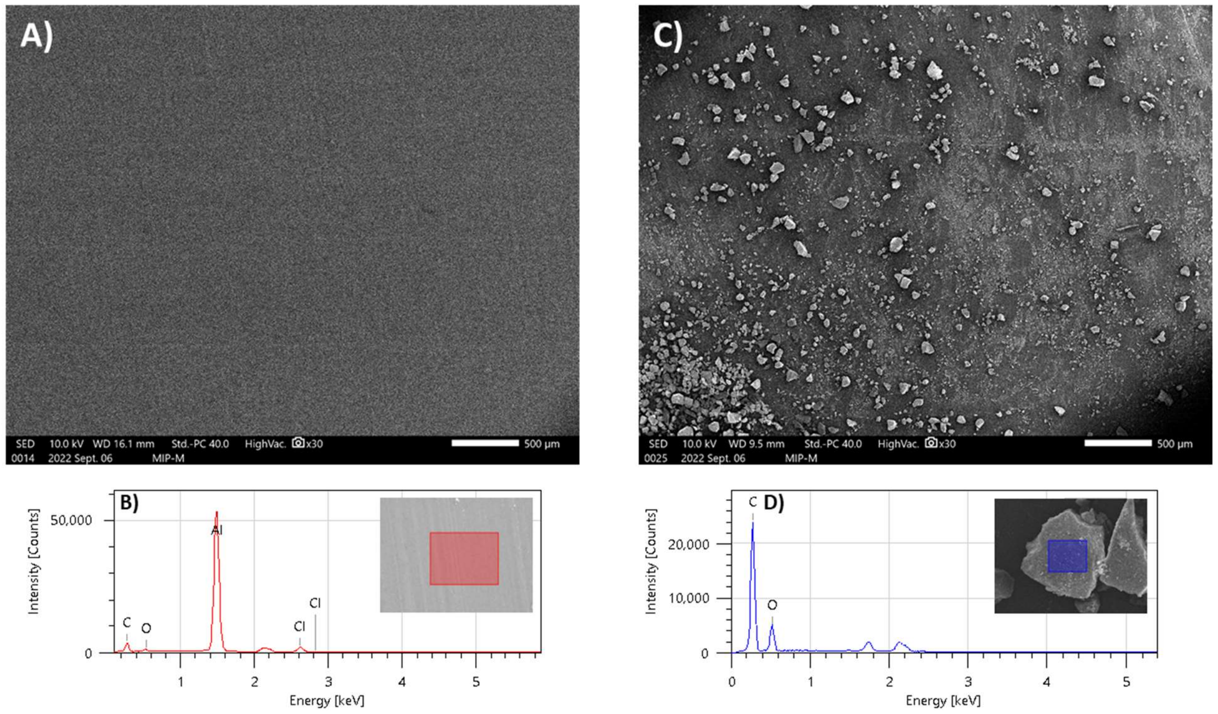

Figure S6. SEM images of the (A) Al-PVC layer and (C) MIP-based receptor layer. EDX analysis of the (B) Al-PVC layer and (D) deposited MIP particle.

References

1. Lorenzo, R.A.; Carro, A.M.; Alvarez-Lorenzo, C.; Concheiro, A. To Remove or Not to Remove? The Challenge of Extracting the Template to Make the Cavities Available in Molecularly Imprinted Polymers (MIPs). *International Journal of Molecular Sciences 2011, Vol. 12, Pages 4327-4347* **2011**, *12*, 4327–4347, doi:10.3390/IJMS12074327.

2. Jawaid, S.; Talpur, F.N.; Afridi, H.I.; Nizamani, S.M.; Khaskheli, A.A.; Naz, S. Quick Determination of Melamine in Infant Powder and Liquid Milk by Fourier Transform Infrared Spectroscopy. *Analytical Methods* **2014**, *6*, 5269–5273, doi:10.1039/c4ay00558a.
